# HDAC4 promotes nasopharyngeal carcinoma progression and serves as a therapeutic target

**DOI:** 10.1038/s41419-021-03417-0

**Published:** 2021-02-01

**Authors:** Chun Cheng, Jun Yang, Si-Wei Li, Guofu Huang, Chenxi Li, Wei-Ping Min, Yi Sang

**Affiliations:** 1 Jiangxi Key Laboratory of Cancer Metastasis and Precision Treatment, Department of Center Laboratory, The Third Affiliated Hospital of Nanchang University, Nanchang, 330008 China; 2 Department of Oncology, Tongji Huangzhou Hospital of Huazhong University of Science and Technology, Hubei, People’s Republic of China; 3 Department of Surgery, Pathology and Oncology, University of Western Ontario, London, N6G5H5 Canada

**Keywords:** Targeted therapies, Cell invasion

## Abstract

Histone deacetylases (HDACs) are involved in tumor progression, and some have been successfully targeted for cancer therapy. The expression of histone deacetylase 4 (HDAC4), a class IIa HDAC, was upregulated in our previous microarray screen. However, the role of HDAC4 dysregulation and mechanisms underlying tumor growth and metastasis in nasopharyngeal carcinoma (NPC) remain elusive. Here, we first confirmed that the HDAC4 levels in primary and metastatic NPC tissues were significantly increased compared with those in normal nasopharyngeal epithelial tissues and found that high HDAC4 expression predicted a poor overall survival (OS) and progression-free survival (PFS). Functionally, HDAC4 accelerated cell cycle G1/S transition and induced the epithelial-to-mesenchymal transition to promote NPC cell proliferation, migration, and invasion in vitro, as well as tumor growth and lung metastasis in vivo. Intriguingly, knockdown of N-CoR abolished the effects of HDAC4 on the invasion and migration abilities of NPC cells. Mechanistically, HDAC3/4 binds to the E-cadherin promoter to repress E-cadherin transcription. We also showed that the HDAC4 inhibitor tasquinimod suppresses tumor growth in NPC. Thus, HDAC4 may be a potential diagnostic marker and therapeutic target in patients with NPC.

## Introduction

Nasopharyngeal carcinoma (NPC), originating from the nasopharynx epithelium, is highly prevalent in East and Southeast Asia, particularly in Southern China^1^. NPC is a poorly differentiated cancer and has the highest metastasis rate among head and neck cancers^2^. Through the combined use of intensity-modulated radiotherapy (IMRT), concurrent chemoradiotherapy, and magnetic resonance imaging (MRI)^3^, locoregional control has steadily improved. However, distant metastasis remains the major cause of treatment failure in patients with NPC^4^. Furthermore, the molecular mechanisms underlying NPC growth and metastasis remain largely unknown. Therefore, effective therapeutic strategies are needed to treat NPC patients.

In our previous study, we used a microarray containing whole human transcription factors to screen a pair of NPC primary tumor tissues and metastatic tissues in lymph nodes (LNs) and two cell lines (S18 and S26) derived from the NPC cell line CNE2 with high and low metastatic abilities, respectively^5^. TEL2 was one of the most altered transcription factors among the downregulated genes^2,5^; however, the upregulated genes were not explored in our previous study. Importantly, histone deacetylase 4 (HDAC4) expression increased simultaneously in LN metastases compared with primary tissues and in S18 cells compared with S26 cells in these microarray results.

Based on sequence homologies, the 18 human HDACs are classified into four groups: class I, class II, class III, and class IV HDACs^6^. HDAC4 is a member of the class IIa HDACs and is similar to the other class IIa members (HDAC5, HDAC7, and HDAC9), which regulate gene expression by mediating different signaling pathways^7,8^. Recently, accumulating evidence has shown that HDAC4 overexpression is correlated with the development and progression of cancers, including multiple myeloma, prostate cancer, breast cancer, colon cancer, glioblastoma, and esophageal cancer^9–14^. Elevated HDAC4 expression plays an oncogenic role via different mechanisms that depend on the cellular context and activated downstream signaling pathways^8^. For example, HDAC4 inhibits HOXB13 transcription to affect cell growth in androgen receptor (AR)-negative prostate cancers^15^, but the HDAC4-RelB-p52 complex maintains a repressive chromatin state around the proapoptotic genes Bim and BMF and regulates multiple myeloma survival and growth^16^. However, the function of HDAC4 and underlying molecular mechanism in NPC are poorly understood.

In the present study, we elucidated the function and underlying molecular mechanisms of HDAC4 in NPC. Our data showed that HDAC4 promotes NPC cell proliferation, invasion, and migration in vitro and tumor growth and lung metastasis in vivo. In addition, HDAC4 promotes the cell cycle and induces the epithelial-to-mesenchymal transition (EMT). Knockdown of N-CoR abolished the effects of HDAC4 on the invasion and migration abilities of NPC cells. Finally, the HDAC4 inhibitor tasquinimod was used to target NPC cells with high HDAC4 expression, suggesting a promising therapeutic strategy.

## Materials and methods

### Cell lines and reagents

Four NPC cell lines (S26, S18, 6-10B, and 5-8F) were cultured in Dulbecco’s modified Eagle’s medium (DMEM; Gibco; Thermo Fisher Scientific, Inc., Waltham, MA, USA) supplemented with 10% fetal bovine serum (Gibco; Thermo Fisher Scientific, Inc.) and maintained at 37 °C in an incubator containing 5% CO_2_. The normal nasopharyngeal epithelial cell line (NP69) was grown in defined-KSFM medium supplemented with epidermal growth factor (EGF) (Invitrogen, Carlsbad, USA). The five cell lines were obtained from Sun Yat-sen University Cancer Center (SYSUCC)^2^. S18 and S26 were derived from the NPC cell line CNE2 with high and low metastatic ability, respectively. 5-8F and 6-10B were derived from the NPC Sune1 cell line with high and low metastatic ability, respectively^4^. HEK-293T cells were obtained from the ATCC and cultured in DMEM (Gibco; Thermo Fisher Scientific, Inc., Waltham, MA, USA). All of the cells were authenticated using short-tandem repeat profiling, tested for mycoplasma contamination, and cultured for <2 months.

### Plasmids

The full-length CDSs of human HDAC4 was cloned into the pSin-puro vector. shRNAs were established using the Sigma-Aldrich shRNA system (Merck KGaA) according to the manufacturer’s protocols. Scr shRNA (with no known targets in the human genome) had the sequence 5′-GGGCGAGGAGCTGTTCACCG-3′, and the oligonucleotides for human HDAC4 shRNA#1 and #2 were 5′-GTTACAAGAATTTGTCCTCAA-3′ and 5′-CGACTCATCTTGTAGCTTATT-3′, respectively. All the recombinant plasmids were verified by DNA sequencing (Ruiboxingke Biotech Co., Ltd., Beijing, China).

### Antibodies

Human anti-HDAC4 (cat. no. 7628) and anti-HDAC3 (cat. no. 85057) were obtained from Cell Signaling Technology. Human anti-N-CoR (cat. no. ab24552) was obtained from Abcam. Anti-β-actin (cat. no. 60008-1) was obtained from ProteinTech Group, Inc. (Chicago, IL, USA). The epithelial–mesenchymal transition (EMT) Antibody Sampler Kit (anti-E-cadherin, anti-N-cadherin, anti-Slug, and anti-Snail) (cat. no. 9782), anti-Cyclin D1 (cat. no. 2978), anti-CDK4 (cat. no. 12790), and anti-CDK6 (cat. no. 13331) were obtained from Cell Signaling Technology. Human anti-HDAC4 (cat. no. sc-46672) was obtained from Santa Cruz Biotechnology for ChIP analysis.

### Stable lines

For the knockdown of HDAC4 in 5-8F and S18 cells, 3 μg of shRNA-Scr or 3 μg of shRNA-HDAC4#1 or #2 was co-transfected with 3 μg of pMD2.G and 3 μg of psPAX2 into 293T cells for 48 h using Lipofectamine 2000. The recombinant viruses were subsequently collected and added to S18 and 5-8F cells, which were then cultured with 8 μg/ml of polybrene for 24 h. 5-8F and S18 cells were infected with recombinant viruses at a multiplicity of infection (MOI) of 10. For the overexpression of HDAC4 in S26 and 6-10B cells, 3 μg of pSin-puro delivering HDAC4 or 3 μg of the empty vector was co-transfected with 3 μg of pMD2.G and 3 μg of psPAX2 into HEK-293T cells for 48 h using Lipofectamine 2000. The recombinant viruses were subsequently collected and added to S26 and 6-10B cells, which were then cultured with 8 μg/ml of polybrene for 24 h. S26 and 6-10B cells were infected with recombinant viruses at an MOI of 5. The stable lines were selected with 1 μg/ml of puromycin for 2 weeks.

### RNA extraction and qRT-PCR

These procedures were performed as previously described^5,17^. Briefly, total RNA was isolated using TRIzol^®^ reagent (Invitrogen; Thermo Fisher Scientific, Inc.) according to the manufacturer’s instructions. First-strand cDNA was synthesized via reverse transcription using the Revert Aid^TM^ First Strand cDNA Synthesis Kit (cat. no. 6210A; TaKaRa Bio, Inc., Otsu, Japan). Subsequently, qRT-PCR was performed using a CFX96 Real-Time PCR Detection System (Bio-Rad Laboratories, Inc., Hercules, CA, USA) and SYBR^®^ Green mix (Tiangen Biotech Co., Ltd., Beijing, China). The qRT-PCR thermal cycling conditions were initiated using a denaturation step at 95 °C for 15 min and comprised 40 cycles (denaturation at 95 °C for 15 s, annealing at 60 °C for 30 s and elongation at 72 °C for 30 s). The amplification products were analyzed using the 2^−∆∆Cq^ method^18^, and the expression levels in each sample were calibrated to those of the housekeeping gene GAPDH. The primers employed for amplifying HDAC4, E-cadherin, N-cadherin, Snail, Slug, and GAPDH are listed in Supplementary Table 1.

### RNA interference

An effective siRNA oligonucleotide targeting N-CoR, 5′-AAUGCUACUUCUCGAGGAAACA-3′, was synthesized by Guangzhou RiboBio Co., Ltd. (Guangzhou, China). Approximately 2.5 × 10^5^ NPC cells/well were seeded in 6-well culture plates the day before transfection. Transfection was performed according to the manufacturer’s instructions, and the cells were transfected with 50 nM of siRNA using Lipofectamine RNAiMAX transfection reagent (Invitrogen; Thermo Fisher Scientific, Inc.).

### Cell proliferation assay

In vitro cell proliferation was assessed using the Cell Counting Kit-8 (CCK8) assay. For cell proliferation, the cells were seeded in 96-well plates at a density of 1000 cells/well and incubated for 1, 2, 3, 4, or 5 days; 10 µl of CCK8 reagent (Beyotime Institute of Biotechnology, Haimen, China) was then added to each well, and the plate was incubated for 1.5 h at 37 °C. The absorbance value (optical density) of each well was measured at 450 nm using an iMark microplate reader (Bio-Rad Laboratories, Inc.).

### Colony formation assay

The cells in this study were plated in six-well culture plates at a density of 5 × 10^2^ cells/well. Each group included three wells. The cells were incubated for 15 days at 37 °C, washed twice with PBS, incubated with methyl alcohol for 15 min, and stained with 0.1% crystal violet for 60 min. Clusters were assessed by light microscopy, and each cluster containing ≥50 cells was counted as a colony.

### Transwell assays

For the Transwell migration assay, 200 μl of serum-free DMEM containing either 4.0 × 10^4^ (S18 and 5-8 F) or 8 × 10^4^ (S26 and 6-10B) cells was added to cell culture inserts with an 8-μm microporous filter without an extracellular matrix coating (BD Biosciences, Franklin Lakes, NJ, USA). DMEM containing 10% fetal bovine serum (FBS; Gibco; Thermo Fisher Scientific, Inc.; cat. no. 10270-106) was then added to the bottom chamber. After 24 h of incubation at 37 °C in 5% CO_2_, the cells on the lower surface of the filter were fixed, stained and examined under a light microscope. The number of migrated cells in three random optical fields (magnification, ×10) from triplicate filters was averaged. For the Transwell invasion assay, 8.0 × 10^4^ (S18 and 5-8F) or 1.6 × 10^5^ (S26 and 6-10B) cells resuspended in 200 μl serum-free DMEM were added to cell culture inserts, which contained 8-μm microporous filters and were coated with Matrigel (BD Biosciences; cat. no. 354480). DMEM containing 10% FBS was then added to the bottom chamber. After 24 h of incubation at 37 °C in 5% CO_2_, the cells on the lower surface of the filter were fixed with methyl alcohol for 15 min at room temperature, stained with 0.1% crystal violet for 60 min at room temperature, and examined under a light microscope. The number of invading cells in three random optical fields (magnification, ×100) from triplicate filters was averaged.

### Cell cycle analysis

These procedures were performed as previously described^19^. Briefly, cells were harvested and fixed with 70% ethanol at 4 °C for 1 h overnight. Next, the cells were stained with PI/RNase and staining buffer at 4 °C for 30 min, followed by analysis using a flow cytometer (BD Biosciences, USA).

### Western blotting and immunoprecipitation

Western blotting procedures were performed as described previously^20,21^. Briefly, cells were collected and lysed in RIPA buffer (150 mM NaCl, 0.5% EDTA, 50 mM Tris, 0.5% NP40) at 4 °C for 30 min and centrifuged for 20 min at 12,000 rpm at 4 °C. The lysates were obtained, and the protein concentration was determined using the BCA method. Protein extracts were separated by 10% SDS-PAGE at a voltage of 120 V for 2 h at room temperature, transferred to PVDF membranes with an electrical current of 350 mA at 4 °C for 2 h, and blocked with 5% non-fat milk for 1 h at room temperature. The membranes were probed with antibodies against HDAC4, E-cadherin, N-cadherin, Snail, Slug, and β-actin. The blots were incubated with peroxidase-conjugated secondary antibodies against rabbit (1:20,000; 401BW, Promega Corporation, Madison, WI, USA) and mouse (1:20,000; W402B, Corporation, Madison, WI, USA), detected using an ECL chemiluminescence system (cat. no. P0018F; Beyotime, China) and exposed to radiographic film (Carestream, Catalog Number 6535876). To analyze the endogenous interaction among HDAC4, N-CoR, and HDAC3 using immunoprecipitation, the clarified supernatants were first incubated with anti-HDAC4, anti-N-CoR, and anti-HDAC3 for 2 h at 4 °C. Next, protein A/G-agarose was added from 2 h to overnight, and the precipitates were washed four times with RIPA buffer and analyzed by western blotting.

### Luciferase assay

The assay was carried out as described previously^17^. Briefly, 6-10B cells were seeded in 12-well plates at a density of 3 × 10^5^ cells/well and transfected with 0.8 μg of a promoter-luciferase reporter plasmid. The cells were co-transfected with 8 ng of Renilla luciferase plasmid as an internal control. After transfection for 48 h, the luciferase activity was measured using the Dual-Luciferase Reporter Assay Kit (Promega, Madison, WI, USA). Three independent experiments were performed.

### ChIP assays

These procedures were performed as previously described^2,5,20^ using a ChIP kit (cat. no. 53008, Active Motif, Carlsbad, CA, USA). Briefly, once the cells reached 80% confluence, Complete Cell Fixative Solution (included in the kit) was added to the existing culture medium at room temperature to fix the cells, and then the fixation reaction was stopped by adding Stop Solution (included in the kit) to the culture medium. The cells were collected by centrifugation at 1000 × *g* for 5 min at 4 °C. Subsequently, the nuclear pellet was resuspended in ChIP Buffer (included in the kit). DNA in the cell lysate was sheared using a sonication instrument (Ningbo Scientz Biotechnology Co., Ltd., Ningbo, China) to 200- to 500-bp-long fragments. Total genomic DNA (input) was quantified, and 20 μg of chromatin from each sample was immunoprecipitated overnight at 4 °C with 5 μg of anti-HDAC3, anti-HDAC4, or normal IgG as a negative control. Next, nucleosome complexes were isolated with protein G-agarose beads for 3 h at 4 °C. Bound DNA-protein complexes were eluted, and cross-links were reversed after a series of washes using the washing reagent provided in the ChIP kit. Purified DNA was resuspended in TE buffer. Subsequently, PCR was performed using PrimeSTAR® Max DNA Polymerase (cat. no. R045A, TaKaRa Bio, Inc.). The qRT-PCR thermal cycling conditions comprised a denaturation step at 94 °C for 2 min, followed by 35 cycles of denaturation at 98 °C for 10 s, annealing at 60 °C for 15 s and elongation at 72 °C for 30 s). The primers are listed in Supplementary Table 1.

### Molecular docking

The crystal structures of HDAC1 (PDB: 6Z2J), HDAC2 (PDB: 5IX0), HDAC3 (PDB: 4A69), HDAC4 (PDB: 4CBT), HDAC6 (PDB: 6CED), HDAC7 (PDB: 3ZNR), and HDAC8 (PDB: 5FCW) were obtained from the RCSB Protein Data Bank. The structural formula of the HDAC4 inhibitor tasquinimod was obtained from PubChem Compound. Ligand docking studies were performed using Maestro, *Schrödinger* (v11.9).

### Animal experiments

All the animal studies were performed in accordance with protocols approved by Nanchang University (Nanchang, China). The mice were maintained under specific pathogen-free conditions at a temperature of 20–25 °C and 50–70% humidity, under a light/dark cycle of 12 h, with free access to water and food. In total, 108 4-week-old male athymic nude mice were obtained from the Shanghai Institutes for Biological Sciences, Chinese Academy of Sciences (Shanghai, China). In each animal experiment, the mice were randomly assigned to each group. For subcutaneous injection, 2 × 10^6^ cells were mixed with 0.2 ml of PBS (pH 7.4) and 30% (v/v) Matrigel matrix (BD Biosciences). The suspensions were injected subcutaneously into the flanks of 4-week-old male athymic nude mice, which were monitored over 5 weeks (*n* = 6 per group). For tail vein injection, 3 × 10^6^ cells were resuspended in 200 μl of PBS (Biological Industries, Beit Haemek, Israel) and injected into the lateral tail vein of mice that did not receive a subcutaneous injection of cells (3 × 10^6^ cells/animal). Mice were sacrificed 8 weeks following injection, and nodules were counted. To analyze the antitumor activity of tasquinimod on tumor growth, mice received tasquinimod (10 mg/kg) or vehicle (0.1% DMSO) every 3 days via intragastric administration.

### IHC and histological evaluation

IHC analysis was performed as described previously^5,21^. Briefly, samples were fixed in 10% formalin for 10 h at room temperature and embedded in paraffin. Sections (3-µm thick) were prepared and mounted onto positively charged glass slides. Sections for HDAC4 staining were incubated in 10 mM citrate buffer (pH 6.0) and boiled in a microwave oven for 15 min. The sections were incubated with primary antibodies against HDAC4 at a 1:250 dilution at 4 °C overnight in a humidified container, and isotype-matched IgG was used as a negative control. IHC staining was evaluated by two independent pathologists specializing in NPC. The expression level of HDAC4 was scored using the semiquantitative immunoreactive score (IRS) scale described by Remmele and Stegner^22^. Briefly, the HDAC4 signal was detected in the cytoplasm and nucleus. The staining intensity (SI) for HDAC4 has four classes in NPC tissues: 0, absent; 1, weak; 2, moderate; and 3, strong. The percentage of stained cells was categorized as follows: 0, no staining; 1, 1–10%; 2, 11–50%; 3, 51– 80%; and 4, 81–100% stained cells. The intensity and proportion scores were then multiplied to obtain a final score.

### Study approval

The use of human NPC tissues was reviewed and approved by the Ethical Committee of The Third Affiliated Hospital of Nanchang University (Nanchang, China). Written informed consent was obtained from all the patients. In total, 74 tumor specimens were collected from patients undergoing NPC resection [tumor percentage >80%; median age, 45 years; age range, 25–82 years; male: female ratio, 20:17; clinical staging: I (*n* = 7), II (*n* = 15), III (*n* = 24), and IV (*n* = 28)] between January 2014 and December 2018. Sixteen normal nasopharyngeal epithelium specimens were collected from healthy people (median age, 43 years; age range, 35–73 years; male:female ratio, 5:3) by resection between January 2013 and December 2017. A human NPC tissue microarray was purchased from Outdo Biotech (Shanghai, China). One hundred nineteen NPC samples were obtained without adjacent normal nasopharynx tissues, and written informed consent was obtained from the patients (median age, 47 years; age range, 20–82 years; male:female ratio, 89:30) with resection between January 2010 and December 2011.

### Statistical analysis

All the in vitro experiments were performed at least three times independently, and the data from each experiment were presented as means ± SD or means ± SEM. All the data followed a normal distribution. Statistical analysis between two groups was performed by Student’s *t*-test (two-tailed) with SPSS version 18.0 (IBM Analytics, USA). Spearman’s correlation analysis was used to assess the correlation between HDAC4 expression and the clinical stage/grade of the tumor. A *P* value <0.05 was considered to indicate statistical significance in all the cases (*), and a *P* value <0.01 was considered to indicate strong statistical significance in all the cases (**).

## Results

### HDAC4 expression increases in the primary tissues and LN metastases of NPC, and high HDAC4 levels predict a poor patient prognosis

**Fig. 1:**
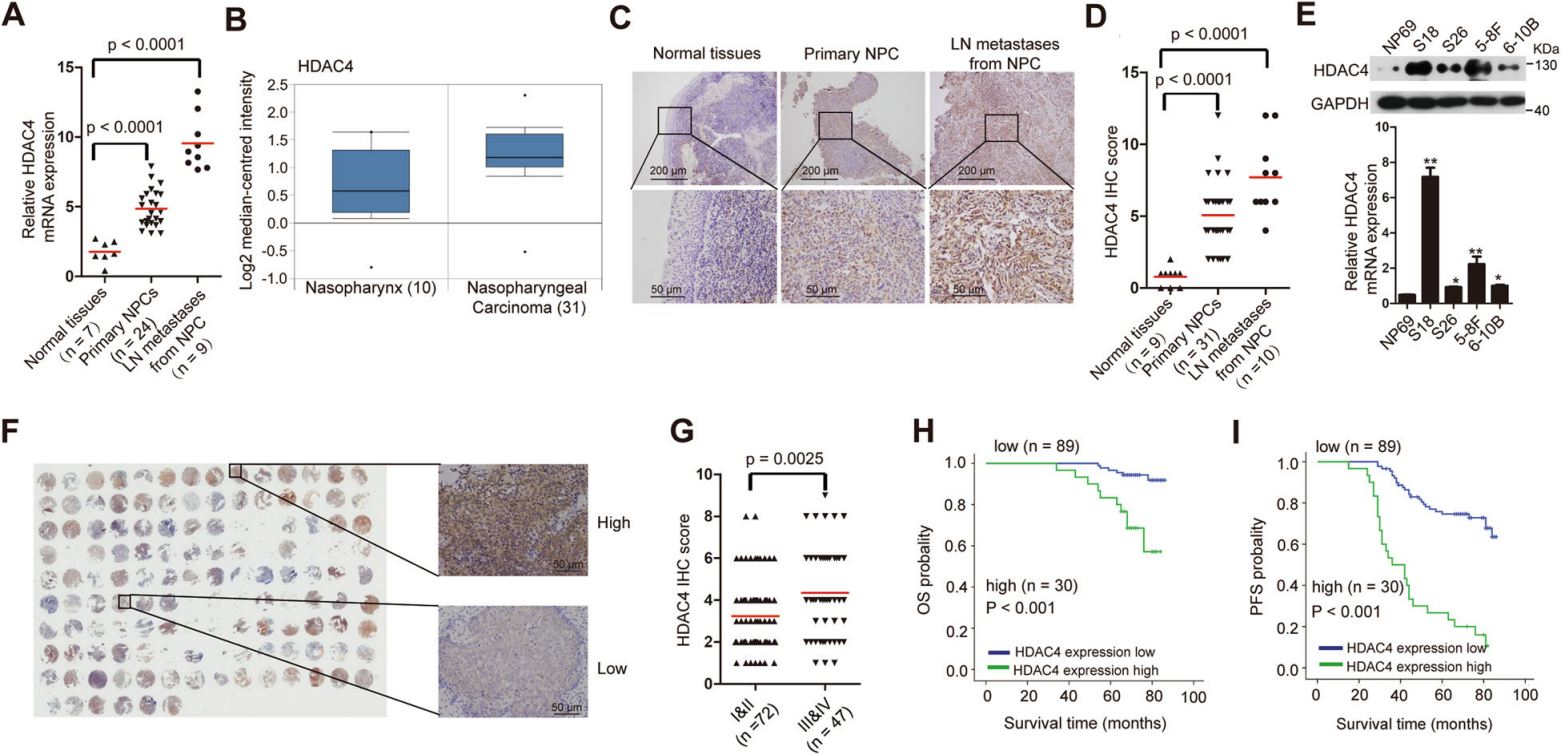
HDAC4 expression increases in primary and metastatic NPC cells, and high HDAC4 expression predicts a poor clinical survival. **A** qRT-PCR analysis of HDAC4 expression in normal nasopharyngeal epithelial tissues (*n* = 7), primary NPC tissues (*n* = 24), and lymph node (LN) metastatic tissues from NPC (*n* = 9). GAPDH was used as an internal control. The bars indicate the mean values. Student’s *t*-test, *P* < 0.0001. **B** Analysis of human HDAC4 expression in normal nasopharyngeal epithelial tissues and NPC tissues using the Oncomine database (http://www.oncomine.org). **C**, **D** IHC staining (**C**) and statistical analysis (**D**) of HDAC4 in normal nasopharyngeal epithelial tissues (*n* = 9), primary NPC tissues (*n* = 31), and lymph node (LN) metastatic tissues from NPC (*n* = 10). Student’s *t*-test, *P* < 0.0001. Scale bar, 200 µm and 50 µm, respectively. **E** The relative mRNA and protein levels of HDAC4 in cell lines were determined by qRT-PCR and western blotting, respectively. GAPDH was used as a loading control. The data are presented as the means ± SD of three independent experiments. Student’s *t*-test, *P < 0.05, **P < 0.01. **F** IHC staining for HDAC4 in 119 clinical NPC samples (left). The images on the right are representative images. **G** HDAC4 protein expression was assessed by IHC analysis of 72 samples with an NPC clinical stage of I–II and 47 with an NPC clinical stage of III–IV. The data are presented as means ± SD. Student’s *t*-test, *P* = 0.0025. Scale bar, 50 µm. **H**, **I** OS (**H**) and PFS (**I**) curves were generated based on the HDAC4 protein expression status of 119 paraffin-embedded NPC tumor tissues, *P* < 0.001.

To further validate the expression level of HDAC4 in NPC according to our microarray data^5^, we first detected its expression in normal nasopharyngeal epithelial tissues, primary NPC tissues, and LN metastases by qRT-PCR. HDAC4 expression was significantly increased in NPC primary tissues and LN metastases compared with that in normal nasopharyngeal epithelial tissues (Fig. 1A). More importantly, HDAC4 expression was higher in LN metastases than in primary tumor tissues. Using the Oncomine database, we found that HDAC4 was also higher in NPC tissues than in normal nasopharyngeal epithelial tissues (Fig. 1B). Notably, immunohistochemistry (IHC) demonstrated that the HDAC4 protein levels were substantially higher in primary tumor tissues and LN metastases than in normal nasopharyngeal epithelial tissues (Fig. 1C, D). Furthermore, we examined the mRNA and protein levels of HDAC4 by qRT-PCR and western blotting in NP69 cells and four other cell lines (S18 and S26; 5-8F and 6-10B, derived from the NPC cell lines CNE2 and Sune1 and exhibiting high and low metastatic ability, respectively)^4^ (Fig. 1E). Both the mRNA and protein levels of HDAC4 were higher in 5-8F and S18 cells than in 6-10B, S26, and NP69 cells. These results suggested that the expression of HDAC4 was upregulated in primary tissues but increased in metastatic tissues.

To evaluate the prognostic value of HDAC4 expression, we performed immunohistochemical (IHC) staining for HDAC4 using a set of tissue microarrays containing 119 NPC samples (Fig. 1F; Supplementary Fig. 1; Supplementary Table 2). IHC analysis revealed that HDAC4 expression was strongly related to the clinical stage of NPC (Fig. 1G). Correlation analysis also demonstrated that higher expression of HDAC4 was positively associated with a more advanced clinical stage of NPC (*r* = 0.244, *P* = 0.008). A similar analysis between HDAC4 and histological classification (World Health Organization, WHO) revealed no such association (*r* = 0.104, *P* = 0.261). Via receiver operating characteristic (ROC) analysis, patients were ranked according to the cutoff values of HDAC4 expression and were divided into two groups (low HDAC4 expression group and high HDAC4 expression group). Kaplan–Meier analysis showed that higher HDAC4 expression was significantly correlated with shorter overall survival (OS) and progression-free survival (PFS) times in patients with NPC (Fig. 1H, I). Moreover, to validate the results of our survival analysis, we applied multivariate Cox regression models, which suggested that HDAC4 was a unique indicator of a poor survival prognosis (Supplementary Table 3). These results confirm that the upregulation of HDAC4 is clinically relevant and may be used as an independent prognostic predictor for NPC patients.

### Overexpression of HDAC4 promotes tumor growth and metastasis in NPC

**Fig. 2:**
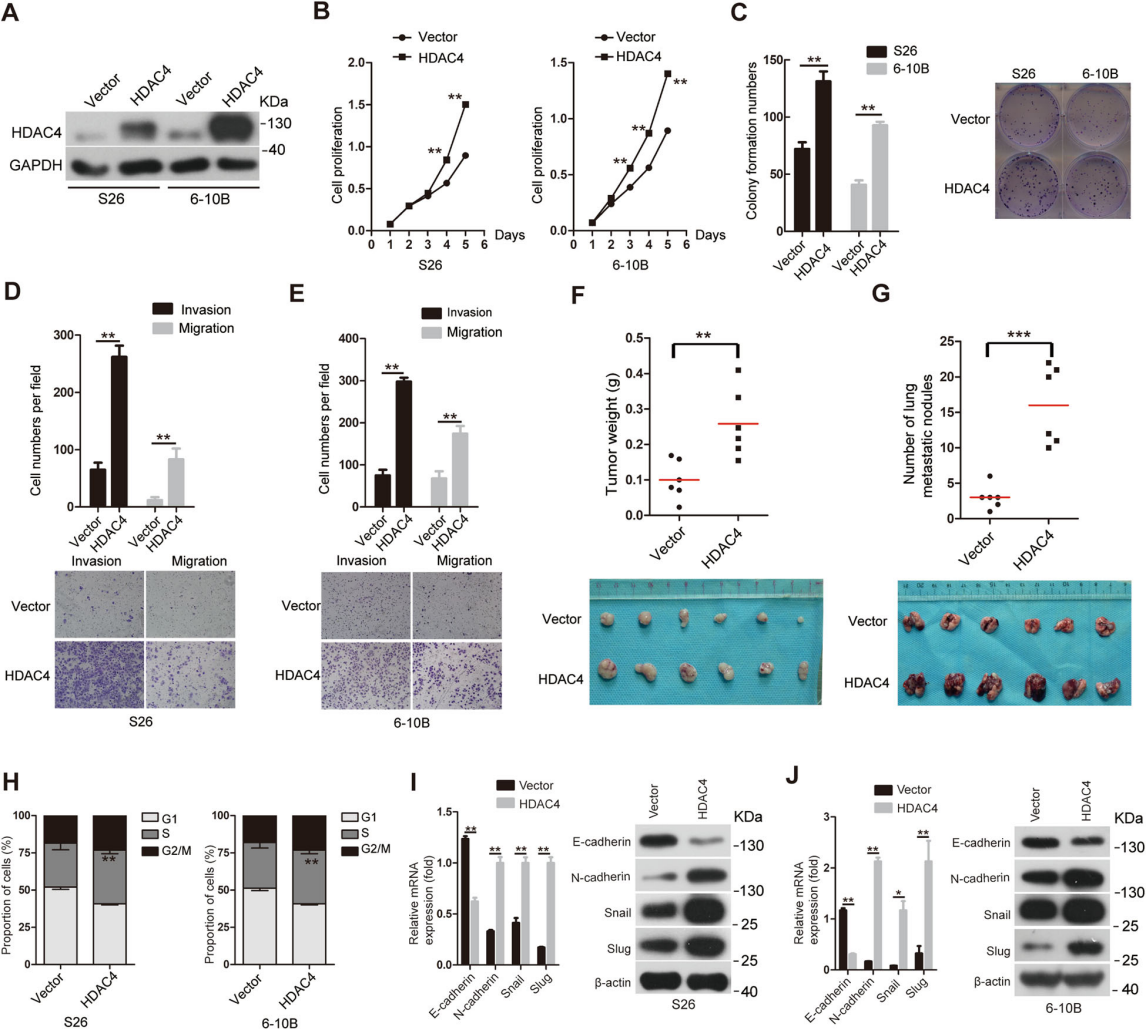
Overexpression of HDAC4 promotes tumor growth and metastasis in NPC. **A** The protein expression levels of HDAC4 were determined in S26 and 6-10B cell lines stably expressing vector or HDAC4 by western blotting. GAPDH was used as a loading control. **B** The proliferation of the indicated cells in vitro was determined by CCK8 assays. The data are presented as the means ± SD of three independent experiments. Student’s *t*-test, **P < 0.01. **C** The colony formation ability of the indicated cells was determined. The images on the right are representative images. The data are presented as the means ± SD of three independent experiments. Student’s *t*-test, **P < 0.01. **D**, **E** Cell migration and invasion were determined in the indicated cells as described in the “Methods” section. The images on the right are representative images in (**D**) and (**E**). The data are presented as the means ± SD of three independent experiments. Student’s *t*-test, **P < 0.01. **F** The weight of orthotopic tumors after subcutaneous injection of 6-10B-vector and 6**-**10B HDAC4 cells. The data are presented as means ± SD. Student’s *t*-test, **P < 0.01. *n* represents the number of nude mice in each group. **G** The number of metastases in the lungs of mice 8 weeks after the tail vein injection of 6-10B-vector or 6-10B HDAC4 cells. The data are presented as means ± SD. Student’s *t*-test, ***P < 0.001. *n* represents the number of nude mice in each group. **H** The cell cycle was investigated in S26-HDAC4 and 6-10B-HDAC4 cells by flow cytometry. The data are presented as the means ± SD of three independent experiments. Student’s *t*-test, **P < 0.01. I, **J** The relative mRNA and protein levels of EMT-related genes were determined by qRT-PCR (left) and western blotting (right), respectively; GAPDH and β-actin were used as loading controls, respectively. The data are presented as the means ± SD of three independent experiments. Student’s *t*-test, *P < 0.05, **P < 0.01.

The above results led us to investigate the function of HDAC4 in NPC. Based on the endogenous expression of HDAC4 in NPC cell lines (Fig. 1E), we constructed stably overexpressing HDAC4 in S26 and 6-10B cells, which have low basal HDAC4 expression (Fig. 2A). The results of CCK8 and colony formation assays showed that the cell proliferation and colony formation abilities were significantly increased after ectopic HDAC4 expression compared with those of control cells (Fig. 2B, C). In addition, we employed Transwell assays by plating cells on inserts coated with or without Matrigel and found that the invasion and migration abilities of cells overexpressing HDAC4 were markedly enhanced in vitro compared with those of control cells (Fig. 2D, E). The above results indicate that NPC cell proliferation, migration, and invasion are regulated by HDAC4 in a manner dependent on its expression. To assess the effect of HDAC4 on NPC growth and metastasis in vivo, we established an animal subcutaneous xenograft model by injecting the 6-10B-vector (an empty control vector) or 6-10B-HDAC4 stable cells subcutaneously into nude mice. The tumor weight indicated that the HDAC4 overexpression group showed increased subcutaneous tumor growth compared with the 6-10B-vector cell group (Fig. 2F). Next, we generated an experimental metastasis model in which stable 6-10B-vector or 6-10B-HDAC4 cells were injected into the lateral tail vein of nude mice and assessed after 8 weeks. As expected, HDAC4 overexpression resulted in a significant increase in lung metastasis in the 6-10B-HDAC4 cell group compared with that in the 6-10B-vector cell group (Fig. 2G). Overall, these results suggest that ectopic expression of HDAC4 may contribute to NPC growth and metastasis.

Next, we investigated whether HDAC4 regulated the cell cycle and EMT to promote NPC growth and metastasis. Flow cytometry assays demonstrated that the S phase showed a significant increase in S26-HDAC4 and 6-10B-HDAC4 cells compared with that in S26-vector and 6-10B-vector cells, respectively (Fig. 2H; Supplementary Fig. 2A, B). To further confirm the results, we analyzed the expression levels of cyclin D1-CDK4/CDK6 complex members, which promote cell progression through the G1/S checkpoint^23^, by western blotting analysis. As expected, the expression of cyclin D1, CDK4 and CDK6 were significantly increased (Supplementary Fig. 2C, D). These results revealed that HDAC4 induced cell cycle progression by promoting the G1/S phase transition. To explore the mechanism underlying HDAC4-mediated promotion of metastasis, we detected markers of EMT using qRT-PCR and western blotting. HDAC4 overexpression upregulated the expression of N-cadherin, Snail and Slug but downregulated the expression of E-cadherin (Fig. 2I, J), suggesting that ectopic expression of HDAC4 is sufficient to induce EMT.

### Knockdown of HDAC4 suppresses cell proliferation, migration, and invasion in vitro and tumor growth in vivo

**Fig. 3:**
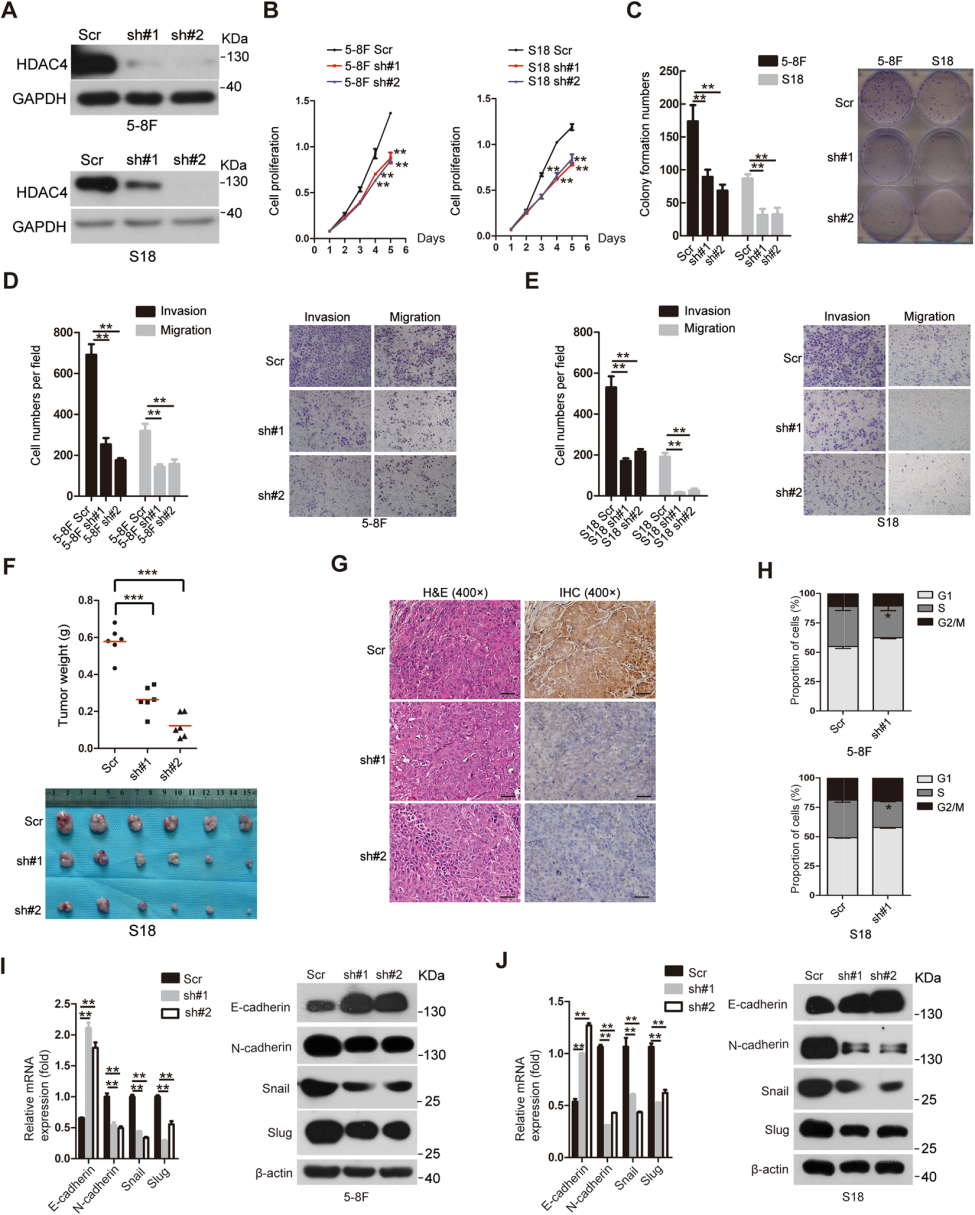
Knockdown of HDAC4 suppresses cell proliferation, migration, and invasion in vitro and tumor growth in vivo. **A** The protein levels of HDAC4 were determined by western blotting in 5-8F and S18 cell lines with or without knockdown. GAPDH was used as a loading control. **B** The proliferation of the indicated cell lines in vitro was determined by CCK8 assays. The data are presented as the means ± SD of three independent experiments. Student’s *t*-test, **P < 0.01. **C** The colony formation ability of the indicated cells was determined. The images on the right are representative images. The data are presented as the means ± SD of three independent experiments. Student’s *t*-test, ^**^P < 0.01. **D** Cell invasion and migration were determined in the indicated stable cell lines as described in the “Methods” section. The images on the right are representative images. The data are presented as the means ± SD of three independent experiments. Student’s *t*-test, **P < 0.01. **E** Cell invasion and migration wer**e** determined in the indicated stable cell lines as described in the “Methods” section. The images on the right are representative images. The data are presented as the means ± SD of three independent experiments. Student’s *t*-test, **P < 0.01. **F** Weight of orthotopic tumors after the subcutaneous injection of S18-Scr cells and S18-shHDAC4#1&2 cells. The bottom panel shows an image of the tumors. The data are presented as means ± SD. Student’s *t*-test, ***P < 0.001. *n* represents the number of nude mice in each group. **G** Representative images of tumors via hematoxylin and eosin (H&E) and immunohistochemical (IHC) staining for HDAC4. Scale bar, 50 µm. **H** The cell cycle was investigated in 5-8F and S18 cells with or without HDAC4 knockdown by flow cytometry. The data are presented as the means ± SD of three independent experiments. Student’s *t*-test, *P < 0.05. **I**, **J** The relative mRNA and protein levels of EMT-related genes were determined by qRT-PCR (left) and western blotting (right), respectively, in the indicated stable lines. GAPDH and β-actin were used as loading controls. The data are presented as the means ± SD of three independent experiments. Student’s *t*-test, **P < 0.01.

To investigate the impact of HDAC4 downregulation on the proliferation and the invasive and migratory abilities of NPC cells, we stably expressed two shRNAs targeting different HDAC4 coding regions (#1 and #2) or a scrambled nontargeting shRNA (Scr) in the 5-8F and S18 cell lines, which have a high basal expression of HDAC4, and examined the protein levels of HDAC4 by western blotting (Fig. 3A). The CCK8, colony formation and Transwell assays were performed and demonstrated that S18 and 5-8F cells with HDAC4 knockdown exhibited reduced proliferation, colony formation, invasion and migration abilities compared with S18-Scr and 5-8F-Scr cells, respectively (Fig. 3B–E). In addition, HDAC4 knockdown in S18 and 5-8F cells resulted in a significant decrease in subcutaneous tumor growth, as shown by measuring the tumor weights (Fig. 3F, G; Supplementary Fig. 3A, B). Taken together, these data indicated that HDAC4 knockdown suppressed the proliferation, invasion and migration abilities of NPC cells in vitro as well as NPC tumor growth in vivo.

We also investigated the cell cycle in stable 5-8F-shHDAC4 and S18-shHDAC4 cell lines; 5-8F-Scr and S18-Scr cells were used as the respective control cells. In contrast to the effects of HDAC4 overexpression, G1 arrest was observed in stable 5-8F-shHDAC4 and S18-shHDAC4 cell lines compared with that in the corresponding control cells (Fig. 3H; Supplementary Fig. 3C, D), and western blotting demonstrated that the protein levels of cyclin D1, CDK4, and CDK6 were decreased after the knockdown of HDAC4 (Supplementary Fig. 3E, F). These results suggested that HDAC4 knockdown induced cell cycle arrest at the G1 phase partly by inhibiting cyclin D1, CDK4, and CDK6 expression. Furthermore, although HDAC4 knockdown abrogated E-cadherin expression, the expression levels of N-cadherin, Snail, and Slug were decreased at the mRNA and protein levels (Fig. 3I, J). Collectively, our results indicate that HDAC4 facilitates tumor growth and metastasis via promoting G1/S transition and EMT in NPC.

### Knockdown of N-CoR abolishes the effects of HDAC4 on the invasion and migration abilities of NPC cells

**Fig. 4:**
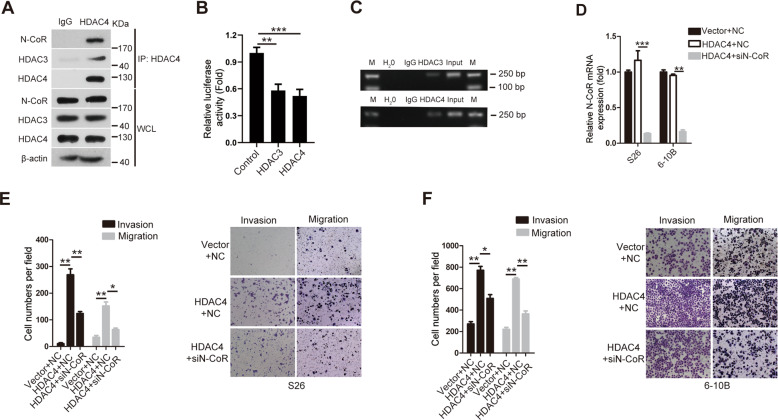
Knockdown of N-CoR abolishes the effect of HDAC4 on the invasion and migration abilities of NPC cells. **A** 6-10B cells were harvested for immunoprecipitation using anti-IgG, anti-HDAC4, anti-N-CoR, and anti-HDAC3 antibodies and then were analyzed by western blotting. β-Actin was used as a loading control. Note: HDAC4 associates with endogenous N-CoR and HDAC3. **B** The luciferase activity of the E-cadherin promoter in 6-10B cells transfected with HDAC3/4 expression plasmids was determined. The Renilla luciferase vector was used as an internal control. **C** ChIP assays determined the binding abilities of HDAC3/4 to the E-cadherin promoter regions in 6-10B cells after transfection with HDAC3/4 expression plasmids. **D** The mRNA level of N-CoR was assessed in the indicated cells by qRT-PCR. GAPDH was used as a loading control. The data are presented as the means ± SD of three independent experiments. Student’s *t*-test, **P < 0.01, ***P < 0.001. **E**, **F** The invasion and migration abilities of the indicated stable cell lines were determined as described in the “Methods” section. The right images are the representative images, and the left images are the statistical results. The data are presented as the means ± SD of three independent experiments. Student’s *t*-test, *P < 0.05, **P < 0.01.

Considering that HDAC4 promotes NPC metastasis, we investigated the molecular partners of HDAC4 to elucidate the mechanism by which HDAC4 regulates NPC metastasis. Because the transcriptional corepressor N-CoR mediates the functional interaction of HDAC4 and HDAC3 (ref. ^24^), we first examined the interaction among HDAC4, N-CoR, and HDAC3 in 6-10B cells. Endogenous coimmunoprecipitation analysis using anti-HDAC3, anti-HDAC4, and anti-N-CoR antibodies showed that HDAC4, N-CoR, and HDAC3 existed in a complex in 6-10B cells (Fig. 4A). As reported previously, HDAC3 binds to the E-cadherin promoter^25,26^. Luciferase assays were then performed to determine the effect of HDAC3/4 on E-cadherin transcription in NPC cells. Ectopic HDAC3 or HDAC4 expression reduced E-cadherin promoter activity in 6-10B cells (Fig. 4B). Furthermore, ChIP confirmed that HDAC3/4 bound to the E-cadherin promoter in 6-10B cells (Fig. 4C). These results suggested that HDAC3/4 was directly bound to the promoter of E-cadherin to repress E-cadherin transcription. Subsequently, we determined whether N-CoR knockdown abolished the effects of HDAC4 on the invasion and migration abilities of NPC cells. S26-HDAC4 and 6-10B-HDAC4 stable cell lines were transfected with or without N-CoR siRNAs (Fig. 4D). The Transwell assays indicated that cotransfection with N-CoR siRNA reversed the increases in the invasion and migration abilities of the S26-HDAC4 and 6-10B-HDAC4 cell lines compared with the transfection of S26-HDAC4 and 6-10B-HDAC4 cells with siRNA-NC (Fig. 4E, F). Collectively, these results show that the HDAC4/N-CoR/HDAC3 complex plays a crucial role in the HDAC4-mediated promotion of invasiveness and migration in NPC cells.

### Tasquinimod suppresses the HDAC4-induced promotion of cell proliferation in vitro

**Fig. 5:**
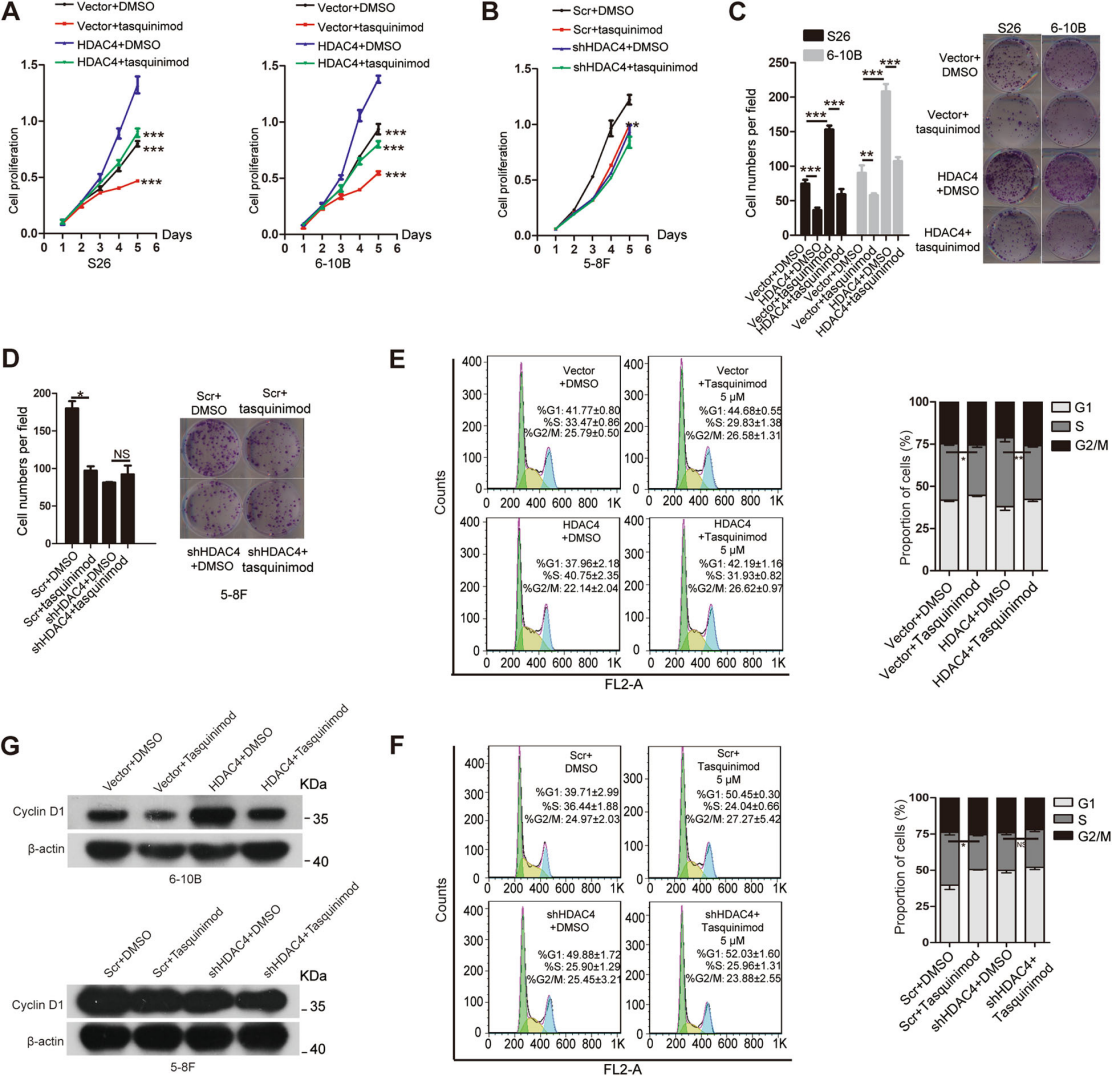
Tasquinimod suppresses the HDAC4-induced promotion of cell proliferation in vitro. **A**–**D** Cell proliferation was analyzed in the indicated stable cell lines by CCK8 (**A**, **B**) and colony formation assays (**C**, **D**) after treatment with 5 μM tasquinimod or vehicle (DMSO) for 48 h. The images on the right (**C**, **D**) are representative images, and the images on the left (**C**, **D**) are the statistical results. The data are presented as the means ± SD of three independent experiments. Student’s *t*-test, *P < 0.05, **P < 0.01, ***P < 0.001. NS: nonsignificant. **E**, **F** Cell cycle was investigated in the indicated cells via flow cytometry after treatment with 5 μM tasquinimod or DMSO for 48 h. The left images are representative images. Each colored area represents cells at different phases of the cell cycle: cells in the G1 phase (green area), S phase (yellow area), and G2/M phase (blue area). The right images are the statistical results. The data are represented as the means ± SD of three independent experiments. Student’s *t*-test. *P < 0.05, **P < 0.01. NS: nonsignificant. **G** The protein levels of cyclin D1 in the indicated cells were determined by western blotting after treatment with 5 μM tasquinimod or DMSO for 48 h. β-Actin was used as a loading control.

Tasquinimod is a high-affinity HDAC4-selective negative allosteric modulator for HDAC4 that can suppress tumor angiogenesis^27^. Considering the above results, we performed CCK8 assays and colony formation to determine whether tasquinimod also inhibits the functional response of NPC cells. Vector cells (S26-vector and 6-10B-vector), HDAC4-overexpressing cells (S26-HDAC4 and 6-10B-HDAC4), and HDAC4-knockdown cells or control cells were treated with 5 μM tasquinimod or DMSO, respectively. Similar to the above results, tasquinimod significantly impaired cell proliferation and colony formation in vector or HDAC4-overexpressing cells compared with those in cells treated with DMSO, as determined by CCK8 and colony assays. In addition, these phenotypes did not significantly change in HDAC4-knockdown cells treated with tasquinimod compared with that in the controls (Fig. 5A–D). Furthermore, the cell cycle in the above cells was analyzed via flow cytometry after treatment with 5 μM tasquinimod for 48 h. As expected, the percentage of cells in S phase was decreased in tasquinimod-treated 6-10B-Vector or 6-10B-HDAC4 cells compared with that in vehicle-treated cells, respectively. However, the percentage of cells in S phase was not significantly decreased in tasquinimod-treated 5-8F shHDAC4 cells compared with that in vehicle-treated cells (Fig. 5E, F). Tasquinimod dramatically decreased cyclin D1 expression, as demonstrated by western blotting (Fig. 5G). Collectively, our results indicated that tasquinimod suppresses HDAC4-induced cell proliferation via G1/S transition in NPC.

Based on the above observations, we speculated whether tasquinimod could also inhibit other HDACs. To test this hypothesis, we characterized the specificity of tasquinimod based on molecular docking studies using the crystal structures of Zn^2+^-dependent HDACs that are accessible online [PDB: 6Z2J; 5IX0; 4A69; 4CBT; 6CED; 3ZNR and 5FCW]. According to the docking results, tasquinimod exclusively bound to Zn^2+^ in the active sites of HDAC4 and HDAC7 among the seven HDACs. Considering the other interactions, such as hydrogen bonds, tasquinimod is predicted to have more affinity with HDAC4, suggesting that tasquinimod mainly affects HDAC4 activity (Supplementary Figs. 4 and 5). However, the effects of tasquinimod on HDAC7 should be further investigated in NPC. In addition, we found no interaction between tasquinimod and HDAC6, and no docking result was obtained using the *Ligand Docking* module of *Schrödinger*.

### Tasquinimod suppresses the HDAC4-induced promotion of cell migration and invasion in vitro

**Fig. 6:**
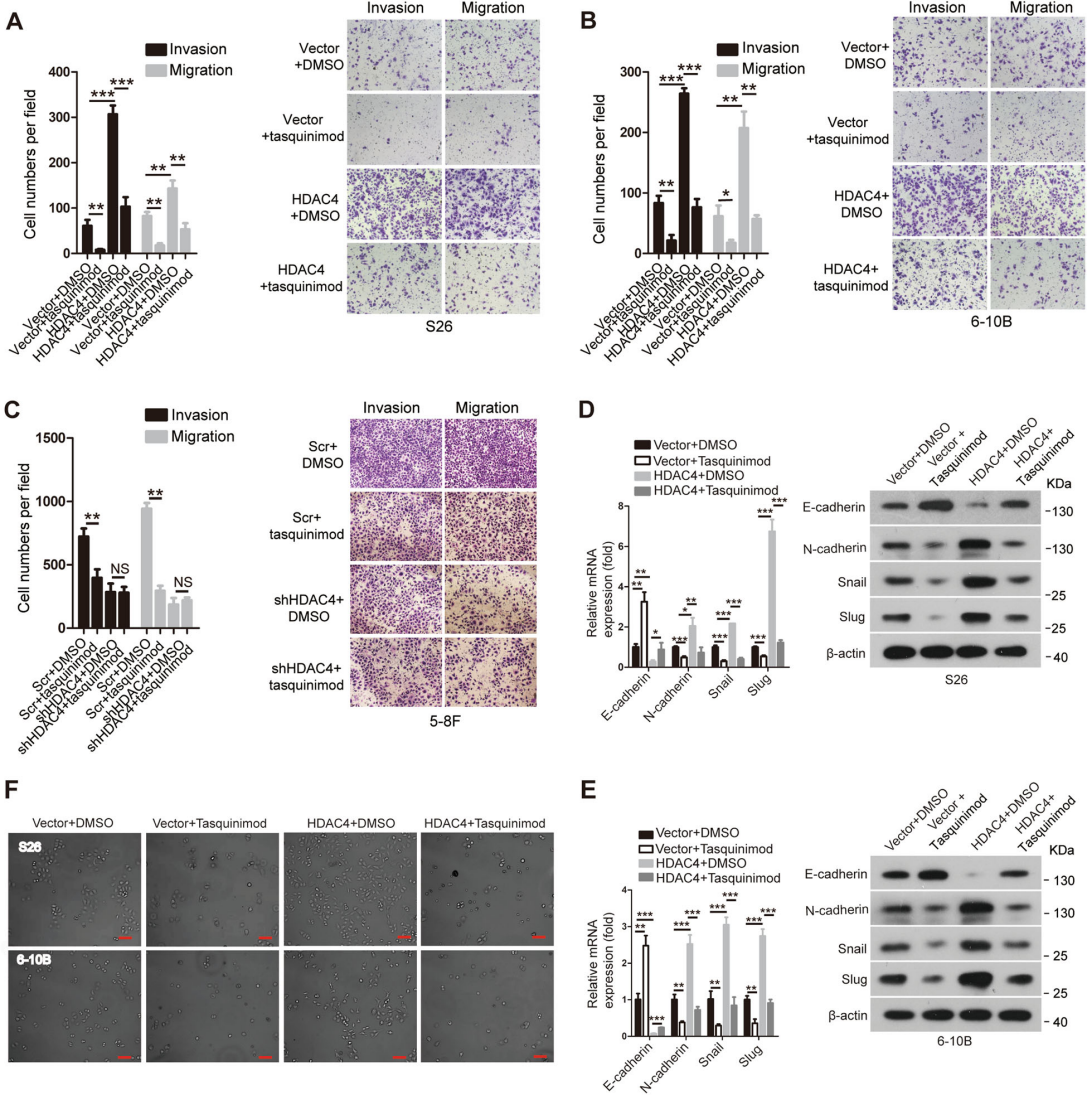
Tasquinimod suppresses the HDAC4-induced promotion of cell migration and invasion in vitro. **A**–**C** The abilities of cell invasion and migration were determined via Transwell assays in the indicated stable cell lines after treatment with 5 μM tasquinimod or vehicle (DMSO) for 48 h. The images on the right are representative images, and the images on the left are the statistical results. The data are presented as the means ± SD of three independent experiments. Student’s *t*-test, *P < 0.05, **P < 0.01, ***P < 0.001. NS: nonsignificant. **D**, **E** The mRNA and protein levels of EMT-related genes were determined by qRT-PCR (left) and western blotting (right), respectively, in the indicated cells treated with 5 μM tasquinimod or DMSO for 48 h. GAPDH and β-actin were used as loading controls. The data are presented as the means ± SD of three independent experiments. Student’s *t*-test, *P < 0.05, **P < 0.01, ***P < 0.001. **F** Representative phase-contrast images of S26 and 6-10B cells after treatment with 5 μM tasquinimod or DMSO for 48 h. The scale bar represents 50 μm.

To investigate the impact of tasquinimod on the invasive and migratory abilities of NPC cells, Transwell assays were performed. The Transwell assays demonstrated that the invasion and migration abilities were lower in vector or HDAC4-overexpressing cells treated with tasquinimod than in cells treated with DMSO, respectively. However, these phenotypes did not significantly change in HDAC4-knockdown cells treated with tasquinimod compared with those in the controls (Fig. 6A–C). Furthermore, tasquinimod abolished HDAC4-mediated EMT, as shown by the detection of EMT markers and observation of phenotypes (Fig. 6D–F), indicating that tasquinimod inhibits HDAC4-induced cell migration and invasion abilities via suppressing the EMT process in NPC.

### Tasquinimod suppresses the HDAC4-induced promotion of tumor growth in vivo

**Fig. 7:**
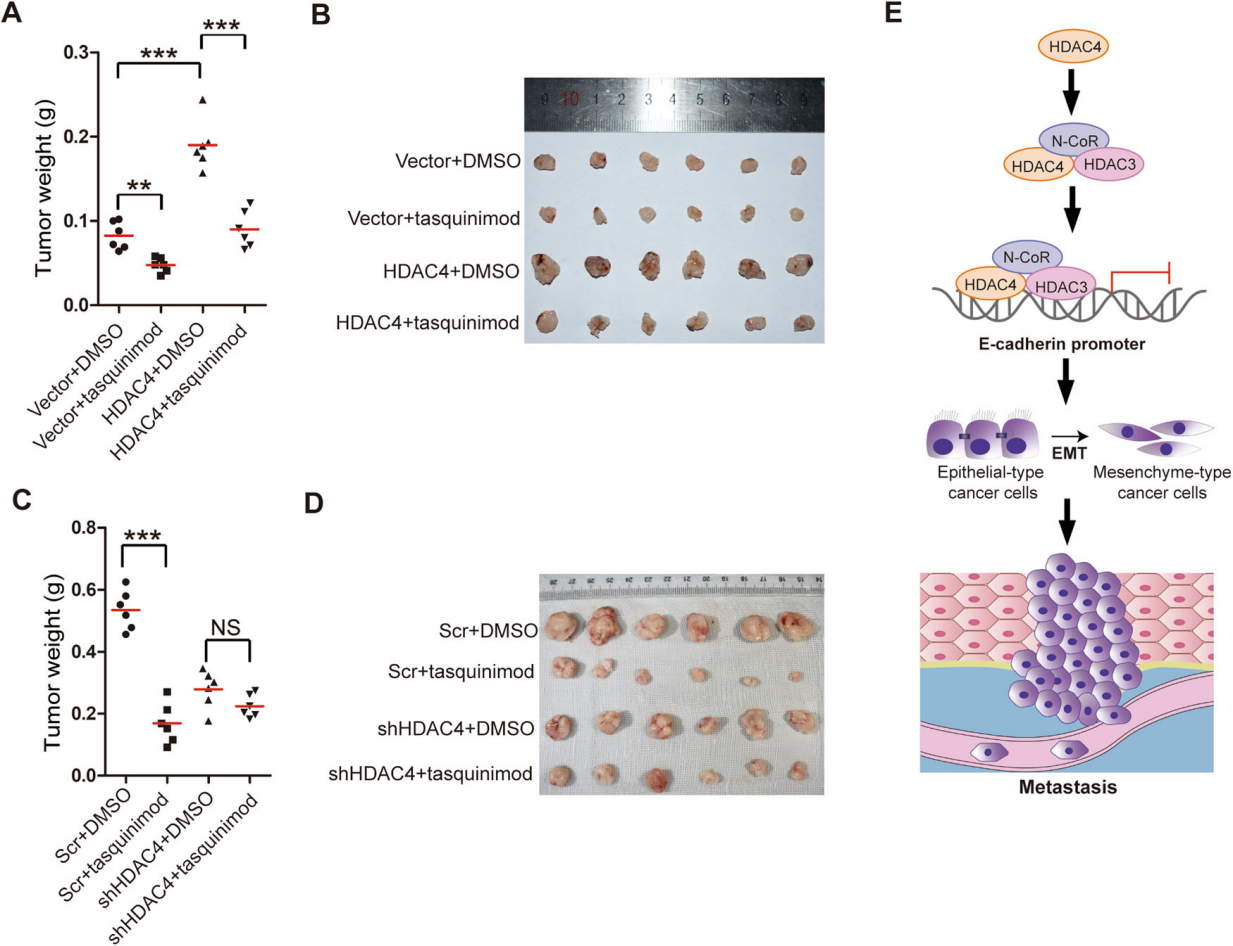
Tasquinimod suppresses the HDAC4-induced promotion of tumor growth in vivo. **A**–**D** The tumor weights (left) in nude mice after the subcutaneous injection of 6-10B-Vector, 6-10B-HDAC4, 5-8F-Scr, or 5-8F-shHDAC4 cells treated with tasquinimod or vehicle (DMSO) (**A**, **C**). The image on the right is the tumor image (**B**, **D**). The data are presented as means ± SD. Student’s *t*-test, **P < 0.01,***P < 0.001. NS: nonsignificant. **E** Proposed model of the role of HDAC4 in promoting tumor metastasis in NPC by binding the promoter of E-cadherin to induce EMT.

Next, we sought to assess the efficiency of tasquinimod therapy in vivo. The weights of orthotopic tumors from 6-10B-Vector, 6-10B-HDAC4, 5-8F-Scr, and 5-8F-shHDAC4 cells were measured after treatment with tasquinimod or vehicle. Compared with the vehicle, tasquinimod inhibited the weight of subcutaneous xenografts established by injecting vector or HDAC4-overexpressing cells (Fig. 7A, B). Furthermore, the reduction in the 5-8F-Scr tumor weight showed a significant difference after tasquinimod treatment compared with that after vehicle treatment. However, the 5-8F-shHDAC4 tumor weights were barely changed after tasquinimod or vehicle treatment (Fig. 7C, D). Given these results, we concluded that tasquinimod significantly reduced tumor growth in NPC.

## Discussion

NPC has the highest metastasis rate among head and neck cancers, and metastasis is the major cause of treatment failure for NPC patients. While previous work has elegantly demonstrated that HDAC4 plays a vital role in cancer development and progression^8^, scant information is available in the literature regarding how HDAC4 is correlated with tumor metastasis. In this study, we demonstrated for the first time that HDAC4 is upregulated in primary and metastatic tissues of NPC. HDAC4 overexpression promoted NPC proliferation, invasion and migration in vitro and enhanced NPC growth and metastasis in vivo. We also showed that HDAC4 overexpression promoted cell cycle G1/S phase transition and EMT. HDAC4 regulates the expression of E-cadherin, N-cadherin, Snail and Slug, and cyclin D1 to promote tumor growth and metastasis in NPC (Fig. 7E).

HDAC4 expression is increased in cancer tissues in various cancers^8^, and our results showed that HDAC4 expression is upregulated not only in primary but also in metastatic tissues of NPC. Therefore, the abnormal expression of HDAC4 demonstrated that it might be closely related to NPC progression. HDAC4 is associated with a poor prognosis and is an independent prognostic factor in esophageal squamous cell carcinoma^14^, and our results showed that a high level of HDAC4 was significantly correlated with a poor prognosis in NPC patients. HDAC4 is an independent prognostic factor in NPC; thus, analyzing the expression of HDAC4 has prognostic value in NPC.

Distant metastasis is the major cause of treatment failure for NPC patients; therefore, understanding the molecular mechanism of NPC is beneficial to the treatment of patients. Previous studies have demonstrated that HDAC4 regulates tumor growth and apoptosis, drug resistance, and cell migration and invasion in colon and hepatocellular carcinoma^8^, and the literature on HDAC4 regulation of cancer metastasis is limited. Moreover, in this study, we provided direct evidence that HDAC4 promotes tumor growth and metastasis in NPC. EMT has been tightly linked with metastasis in diverse types of tumors^28–30^. Our results showed that overexpression or knockdown of HDAC4 increased or decreased the expression of E-cadherin, N-cadherin, Snail and Slug, and may contribute to NPC metastasis. Although HDAC4 promotes the growth of colon cancer cells by suppressing p21 (ref. ^12^), p21 was not significantly altered in our study (data not shown).

The development of specific HDAC isoform inhibitors that suppress HDAC activity to achieve clinical results in cancer therapy is one of the most promising approaches. Moreover, understanding the underlying mechanisms by which these inhibitors kill cancer cells is important to effectively treat cancer patients. Tasquinimod, a small molecule that allosterically binds to the regulatory Zn^2+^ binding domain of HDAC4 (ref. ^27^), prevented the formation of the HDAC4/N-CoR/HDAC3 repression complex by inhibiting the co-localization of N-CoR and HDAC3 (ref. ^27^). Tasquinimod has demonstrated a good safety profile to treat prostate cancer in phase II and III clinical trials^31^ but has yet to be explored therapeutically in NPC. This study found that tasquinimod exhibited potent inhibitory effects on cell proliferation, colony formation, invasion, and migration in vitro and tumor growth in vivo in NPC, suggesting that tasquinimod is a potential candidate for NPC treatment. In addition, molecular docking analysis showed inhibitory activity of tasquinimod against HDAC7, but further studies are necessary to verify this observation. A study indicated that the use of tasquinimod had several adverse effects for the treatment of metastatic castration-resistant prostate cancer (mCRPC), such as anemia, back pain, and insomnia^32^. However, tasquinimod has a well-tolerated safety profile suitable for chronic use, which is an option for men before taxane chemotherapy to delay progression and symptomatic disease^33^. Therefore, toxicity and dosage studies must be performed before treatment in patients with NPC. Research has shown that HDAC inhibitors might act against specific HDACs (isoform-selective inhibitors) and all HDAC types (pan-inhibitors)^34^. Considering the potency and selectivity of HDAC inhibitors, effective inhibitors of HDAC4 warrant further study and development. In addition, the combination with other drugs may be more effective in treating NPC patients. However, further experiments are required to verify this hypothesis.

In conclusion, our present study highlighted an important role of HDAC4 in promoting tumor growth and metastasis in NPC. HDAC4 may serve as a valuable target for treating patients with NPC.

## Supplementary information

Supplementary Figure Legends

Supplementary Tables

Supplementary Fig. 1

Supplementary Fig. 2

Supplementary Fig. 3

Supplementary Fig. 4

Supplementary Fig. 5
