# Supplementary material for: HDAC4 promotes nasopharyngeal carcinoma progression and serves as a therapeutic target
Source: Cell Death Dis. 2021 Feb 1;12(2):137. doi: 10.1038/s41419-021-03417-0 (PMC7862285; doi:10.1038/s41419-021-03417-0)
Supplement: Supplementary file 1 — Supplementary Figure Legends [file 41419_2021_3417_MOESM1_ESM.docx]

**Supplementary Figure Legends**

**Supplementary Fig. 1. S**uitability of HDAC4 antibody for IHC. We used stable cell lines with knockdown of Scr or HDAC4 in 5-8F cells. The cells were collected, fixed and embedded in paraffin, and IHC staining was performed using this antibody. Scale bar, 50 µm.

**Supplementary Fig. 2.** (a, b) Representative images of the cell cycle distribution investigated in the indicated cells by ﬂow cytometry. (c, d) The protein levels of cyclin D1, CDK4 and CDK6 in S26 and 6-10B cells stably expressing vector or HDAC4 were determined by western blotting. β-Actin was used as a loading control.

**Supplementary Fig. 3.** (a, b) Weight of orthotopic tumors after the subcutaneous injection of 5-8F-Scr cells and 5-8F-shHDAC4#1&2 cells. The left represents the statistical results (a), and the right shows an image of the tumors (b). The data are presented as means ± SD. Student’s t-test, ^***^P < 0.001. n represents the number of nude mice in each group. (c, d) Representative images of the cell cycle distribution investigated in the indicated cells by ﬂow cytometry. (e, f) The protein levels of cyclin D1, CDK4 and CDK6 in 5-8F and S18 cells with the knockdown of HDAC4 or Scramble (Scr) were determined by western blotting. β-Actin was used as a loading control.

**Supplementary Fig. 4.** Docking result for inhibitor tasquinimod (green) at the HDAC4 (PDB: 4CBT) (a) and HDAC7 (PDB: 3ZNR) (b) substrate-binding pocket in two-dimensional (2D) and three-dimensional (3D) structures.

**Supplementary Fig. 5.** Docking result for inhibitor tasquinimod (green) at the HDAC1 (PDB: 6Z2J), HDAC2 (PDB: 5IX0), HDAC3 (PDB: 4A69) and HDAC8 (PDB: 5FCW) substrate-binding pocket in two-dimensional (2D) and three-dimensional (3D) structures.
