# Supplementary material for: HDAC4 promotes nasopharyngeal carcinoma progression and serves as a therapeutic target
Source: Cell Death Dis. 2021 Feb 1;12(2):137. doi: 10.1038/s41419-021-03417-0 (PMC7862285; doi:10.1038/s41419-021-03417-0)
Supplement: Supplementary file 2 — Supplementary Tables [file 41419_2021_3417_MOESM2_ESM.docx]

**Supplementary Table 1: Primers used for genes**

| **Primer name** | **Sequence** |
| --- | --- |
| HDAC4-RT-F | 5'-CCTGGGAATGTACGACGCC-3' |
| HDAC4-RT-R | 5'-CCCGTCTTTCCTGCGTAAC-3' |
| E-cadherin-RT-F | 5’-AATAGTGCCTAAAGTGCTGC-3’ |
| E-cadherin-RT-R | 5’-AGACCCACCTCAATCATCCT-3’ |
| N-cadherin-RT-F | 5’-ATCCTACTGGACGGTTCG-3’ |
| N-cadherin-RT-R | 5’-TTGGCTAATGGCACTTGA-3’ |
| Snail-RT-F | 5’-AGTTTACCTTCCAGCAGCCCTAC-3’ |
| Snail-RT-R | 5’-AGCCTTTCCCACTGTCCTCAT-3’ |
| Slug-RT-F | 5’-AAGCATTTCAACGCCTCCAAA-3’ |
| Slug-RT-R | 5’-GGATCTCTGGTTGTGGTATGACA-3’ |
| N-CoR-RT-F | 5’-ACACCGCAGTATTGTCCAAAT-3’ |
| N-CoR-RT-R | 5’-CACCTGGTTTGTCTTGATGTTCT-3’ |
| GAPDH-RT-F | 5’-ACAGTCAGCCGCATCTTCTT-3’ |
| GAPDH-RT-R | 5’-GACAAGCTTCCCGTTCTCAG-3’ |
| E-cadherin -ChIP-F | 5’-TGGTGGTGTGCACCTGTACT-3’ |
| E-cadherin -ChIP-R | 5’-GACCTGCACGGTTCTGATTC-3’ |

**Supplementary Table 2: Clinical characteristics of 119 NPC patients**

| **Characteristics** | **No. (%)** |
| --- | --- |
| **Age, years** |  |
| Median | 47 |
| Range | 20-82 |
| **Sex** |  |
| Male | 89(74.8) |
| Female | 30(25.2) |
| **Histological classification (WHO)** |  |
| Type II | 15(12.6) |
| Type III | 104(87.4) |
| **Clinical staging** |  |
| I | 15(12.6) |
| II | 57(47.9) |
| III | 31(26.1) |
| IV | 16(13.4) |
| **Death** |  |
| Yes | 16 |
| No | 103 |

**Supplementary Table 3. Univariate and multivariate Cox regression analysis of HDAC4**

| **Variable** | **Univariate Cox regression** | | | **Multivariate Cox regression** | |
| --- | --- | --- | --- | --- | --- |
|  | **All cases** | **HR (95% CI)** | **P-*value*** | **HR (95% CI)** | **P-*value*** |
| **Age** |  |  | 0.148 |  |  |
| ≤47 | 49 | 1 |  |  |  |
| >47 | 70 | 0.434(0.140-1.346) |  |  |  |
| **Sex** |  |  | 0.892 |  |  |
| Male | 89 | 1 |  |  |  |
| Female | 30 | 0.925(0.298-2.868) |  |  |  |
| **Histological classification (WHO)** |  |  | 0.305 |  |  |
| Type II | 15 | 1 |  |  |  |
| Type III | 104 | 25.402(0.053-12213.856) |  |  |  |
| **Clinical staging** |  |  | 0.001 |  | 0.004 |
| Early (I&II) | 72 | 1 |  | 1 |  |
| Late (III&IV) | 47 | 12.452(2.828-54.827) |  | 9.218(2.049-41.469) |  |
| **HDAC4 expression** |  |  | <0.001 |  | 0.008 |
| Low | 89 | 1 |  | 1 |  |
| High | 30 | 6.257(2.258-17.342) |  | 4.002(1.431-11.197) |  |
