# Supplementary figures and images for: HDAC4 promotes nasopharyngeal carcinoma progression and serves as a therapeutic target
Source: Cell Death Dis. 2021 Feb 1;12(2):137. doi: 10.1038/s41419-021-03417-0 (PMC7862285; doi:10.1038/s41419-021-03417-0)

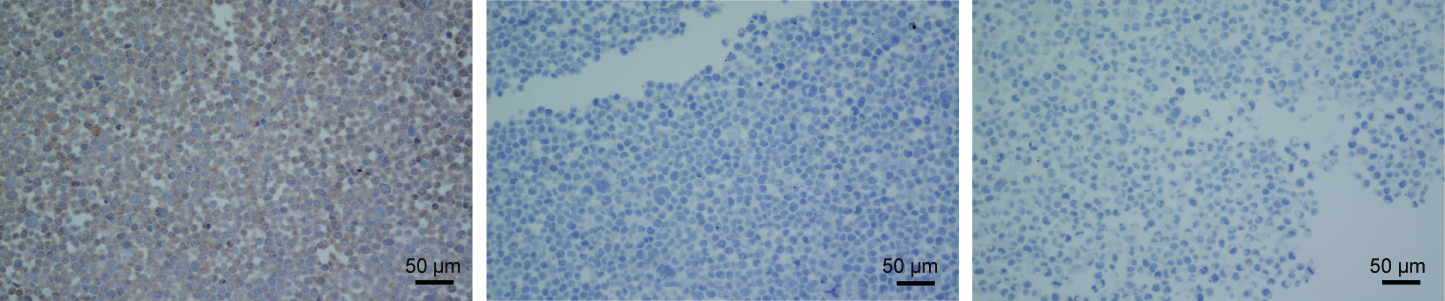

Supplement: Supplementary file 3 — Supplementary Fig. 1 [file 41419_2021_3417_MOESM3_ESM.tif]

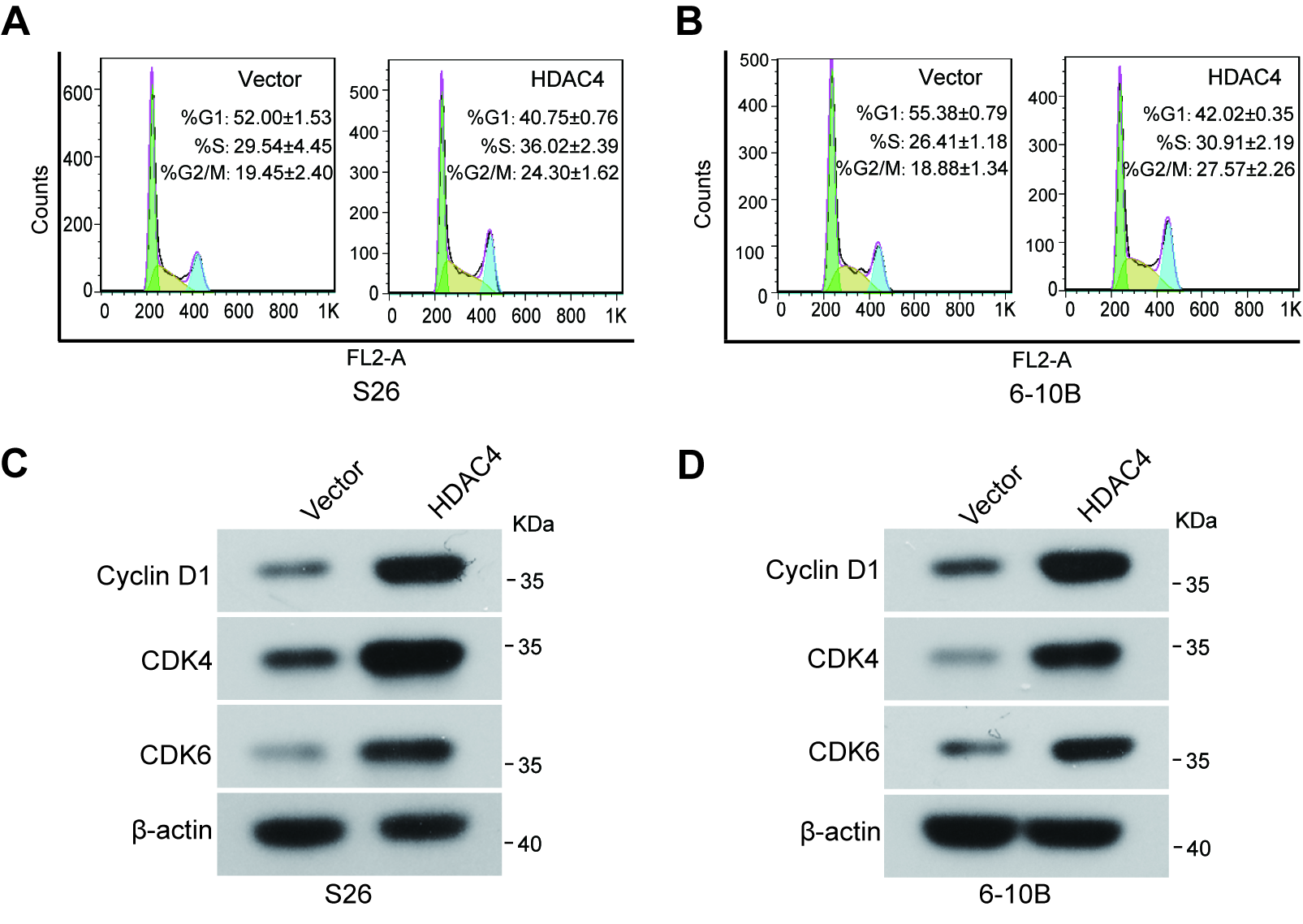

Supplement: Supplementary file 4 — Supplementary Fig. 2 [file 41419_2021_3417_MOESM4_ESM.tif]

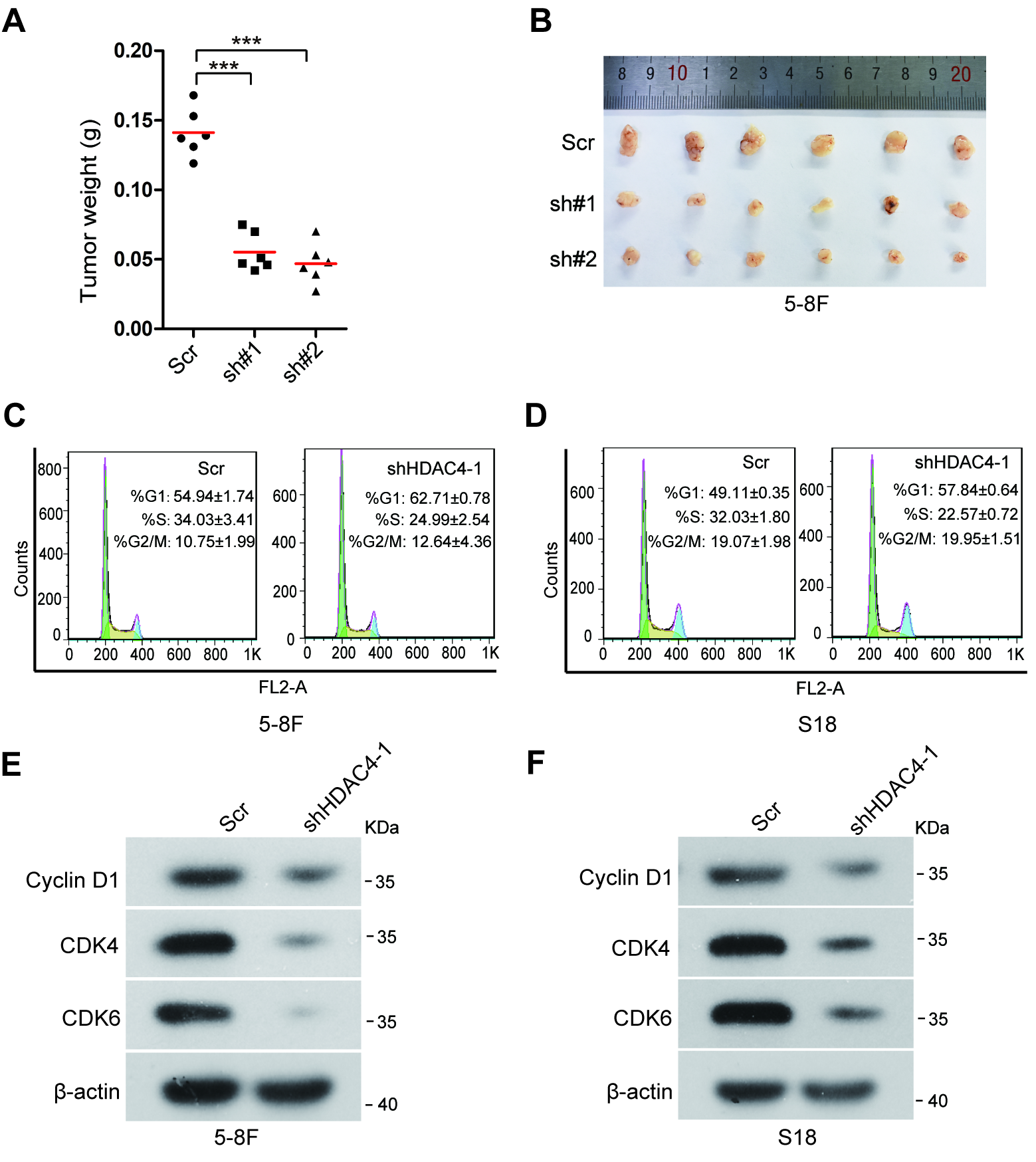

Supplement: Supplementary file 5 — Supplementary Fig. 3 [file 41419_2021_3417_MOESM5_ESM.tif]

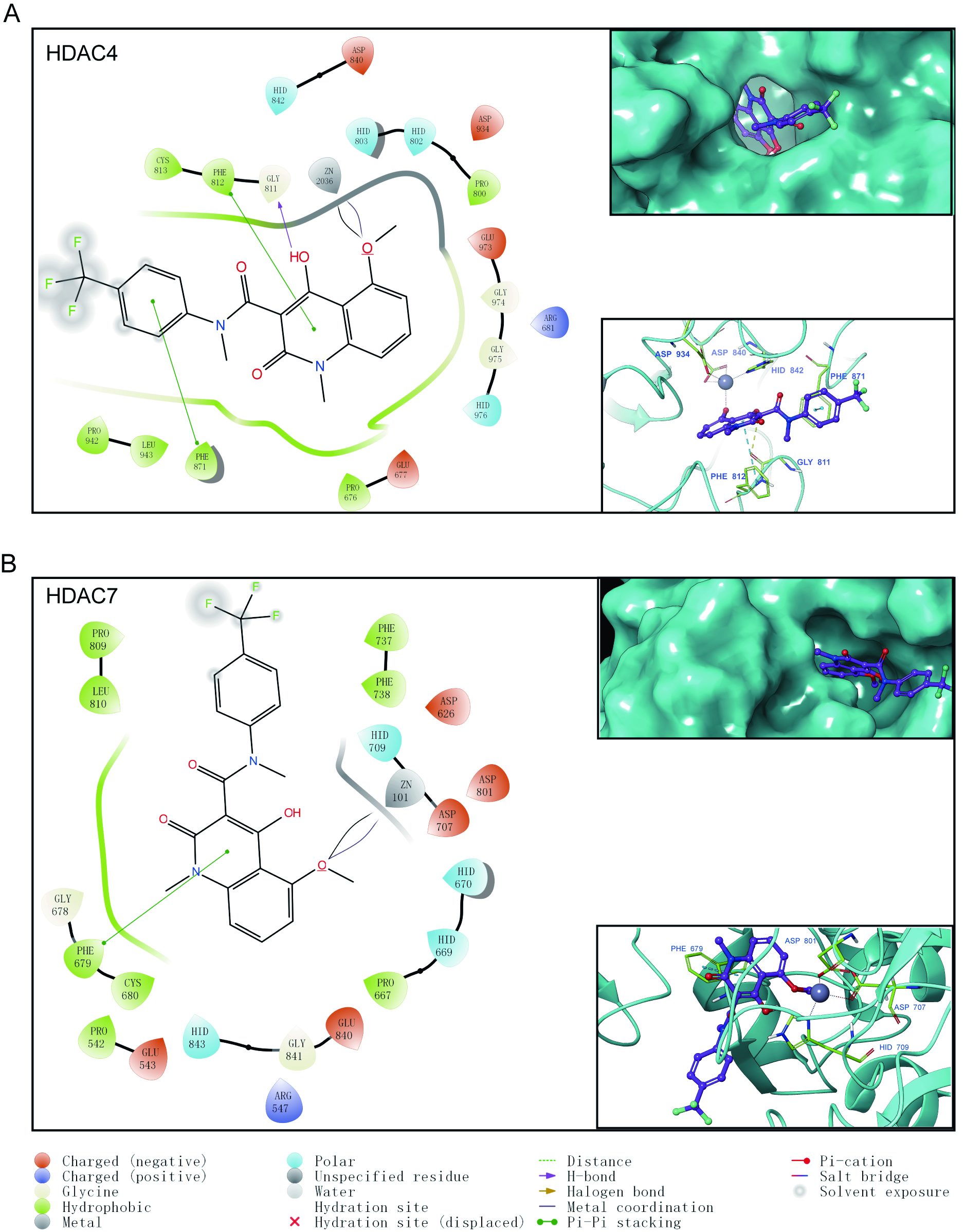

Supplement: Supplementary file 6 — Supplementary Fig. 4 [file 41419_2021_3417_MOESM6_ESM.tif]

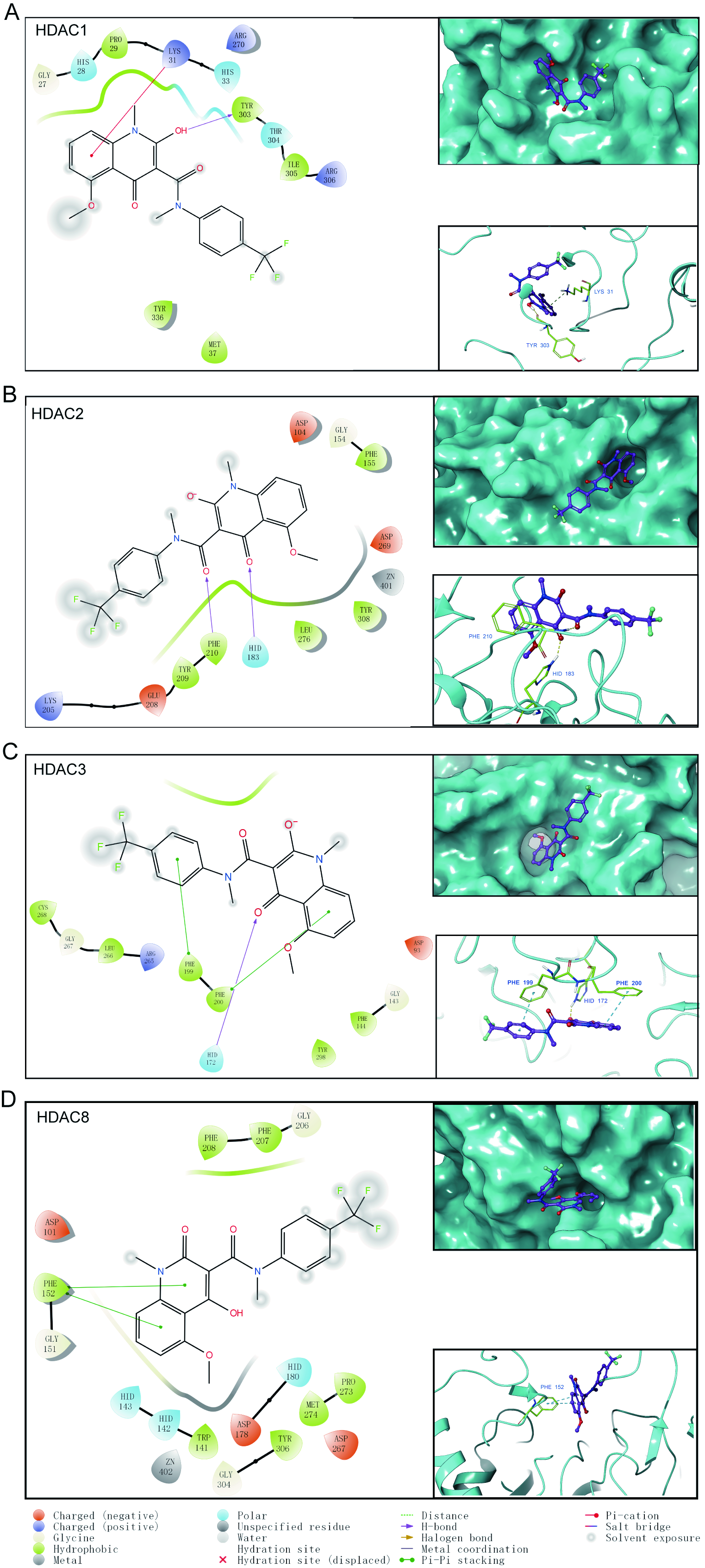

Supplement: Supplementary file 7 — Supplementary Fig. 5 [file 41419_2021_3417_MOESM7_ESM.tif]
